# Gel Strength of Hydrophilic Matrix Tablets in Terms of ***In Vitro*** Robustness

**DOI:** 10.1007/s11095-021-03068-y

**Published:** 2021-06-21

**Authors:** Seyedreza Goldoozian, Valentyn Mohylyuk, Andriy Dashevskiy, Roland Bodmeier

**Affiliations:** 1 College of Pharmacy, Freie Universität Berlin, Kelchstrasse 31, 12169 Berlin, Germany; 2 School of Pharmacy, Queen’s University Belfast, 97 Lisburn Road, Belfast, BT9 7BL UK

**Keywords:** gel strength, HPMC, matrix tablets, mechanical stress, release robustness

## Abstract

**Purpose:**

The purpose of this study was to correlate the gel strength of swollen matrix tablets with their *in vitro* robustness against agitation intensity and applied mechanical forces. Five commercial products, i.e. Glucophage®, Alfuzosin®, Tromphyllin®, Preductal® MR and Quetiapin® formulated as water-soluble/erodible matrix tablets were investigated.

**Methods:**

Effect of agitation speed (50–150 rpm) on drug release, hydration/erosion and gel strength was investigated using USP paddle apparatus II. The gel strength of matrix tablets during dissolution at different conditions was characterized by a texture analyzer.

**Results:**

Commercial tablets formulated with HPMC of higher viscosity, such as K15M or K100M, demonstrated the gel strength in swollen state >0.02 MPa. In this case, the release mechanism was predominantly diffusional and, therefore, not affected by stirring speed and mechanical stress. In contrast, the Quetiapin® matrix tablet, formulated with HPMC K 4 M in amount of approx. 25%, demonstrated the gel strength dropped below 0.02 MPa after 6 h of release. In this case, the drug was predominantly released via erosional mechanism and very susceptible to stirring speed.

**Conclusion:**

Sufficient gel strength of swollen tablets is an important prerequisite for unchanged *in vitro* performance in consideration of mechanical stress.

## Introduction

Hydrophilic matrix tablets remain an important approach to achieve controlled oral drug release. They are formulated using non-cross-linked, water-swellable polymers, e.g. hydroxypropyl methylcellulose (HPMC), that swell rapidly enough to form a continuous ‘gel layer’ surrounding the dry core in order to control the rate of drug release during passage of the matrix through the gastrointestinal (GI) tract.

The behavior of the hydrophilic matrix tablet during release is nowadays well understood. Upon hydration, the aqueous medium penetrates gradually from the surface to the core. Considering the physical state of the drug, zones with dissolved and undissolved drug are distinguished (1). Considering the matrix, the surface of the tablet swells, and an outer gel layer is formed associated with polymer chains relaxation (2,3). Gradual matrix hydration toward the core results in formation of a polymer concentration gradient while three zones, namely, outer gel layer, swollen glassy layer and dry core can be identified. Accordingly, the erosion front (swollen matrix–solvent boundary), diffusion front (solid drug-drug solution boundary) and swelling front (polymer glassy–rubbery transition boundary) were described (1,2). Drug dissolution in the gel and polymer relaxation affect the relative movement of the fronts and, thus, the distance between swelling and diffusion fronts (4,5).

Respectively, the mechanical strength and diffusion rate is decreased through the polymer from the surface towards the core (6,7). With progressing hydration time, the polymer concentration at the gel surface ultimately becomes too low (critical polymer concentration) to withstand shear forces by the surrounding solution. Consequently, the outermost layer of chains starts to detach from the gel surface and slows down further increasing of the swollen layer thickness of the tablet (7). This critical polymer concentration will be constant for each formulation during the entire dissolution process when the shear force environment remains constant (8–10). As polymer disentangles from the erosion front, drug release from matrix tablet occurs via a combination of two mechanisms: drug diffusion through the gel layer and matrix erosion. The drug release mechanism can be considered as relaxation- or diffusion-controlled (Fickian behavior) if the time for polymer chain relaxation is higher than the time for drug diffusion through the polymer or if the diffusion time is higher than relaxation time, respectively. In cases where the relaxation time was approaching the diffusion time, the non-Fickian, or anomalous diffusion, was observed (11–14). The mechanism of drug release is dependent on the movement of particular fronts, especially on the interlay of the erosion and diffusion fronts (1,11,15). The effect of these mechanisms is strongly dependent on the composition and dimensions of tablets, as well as the solubility of the drug and its distribution in the matrix. For example, decrease of drug solubility and slower dissolution rate predetermined the presence of solid drug-particles in the gel layer, resulting in reduced polymer chain entanglement and, consequently, decreased the mechanical strength/ resistance of gel toward erosion (16). It was shown that even freely soluble drugs (e.g. diprophylline with solubility of 235 mg/mL) can be transported by gel-layer (17). The properties and content of the matrix forming polymer are key parameters affecting the processes governing drug release (6,18–22).

The erosion rate depends on the gel strength of the outer gel layer under certain release conditions (10,23,24). Among the different parameters affecting *in vitro* drug release, the hydrodynamic condition (agitation intensity) and mechanical destructive forces play a significant role in drug release from hydrophilic matrices (25,26). However, the *in vivo* drug release can differ from the predicted *in vitro*, due to extensive mechanical forces in the GI tract (26–29). Therefore, the design of a mechanically robust formulation (which can be defined by sufficient gel strength) is a key to achieve predictable *in vivo* plasma concentrations (6).

Among these physiological conditions, hydrodynamic properties (agitation intensity) and mechanical destructive forces within the stomach and intestine have a significant impact on drug release from hydrophilic matrix tablets (30). It has been reported that the tablets can undergo mechanical forces of 2 N or 1.2 N when passing through the human stomach and small intestine, respectively (25,31). These forces can accelerate the erosion and overall release from hydrophilic tablets (26,30,32,33) and *in vivo* drug release can significantly differ from that determined *in vitro* (34). Therefore, formulations with sufficient gel strength can deliver the drug mostly to lower GI segments with greater predictability based on *in vitro* results and less inter-subject variability (6).

Besides hydration and erosion studies, the understanding of gel strength at the gel-solution interface (erosion front) and across the gel layer under dissolution conditions with different agitation can provide a new insight into the robustness of hydrophilic matrix tablets against mechanical stress. Therefore, the aim of this study was to correlate the gel strength of hydrophilic matrix tablets with their *in vitro* mechanical robustness.

## Materials and Methods

### Materials

Five commercial products, namely, Glucophage® XR 500 mg (Merck Serono GmbH, Darmstadt, Germany), Alfuzosin-ratiopharm® uno 10 mg (Ratiopharm GmbH, Ulm, Germany), Tromphyllin® retard 300 mg (Trommsdorff GmbH & Co. KG, Alsdorf, Germany), Preductal® MR 35 mg (Les Laboratories Servier, Gidy, France), and Quetiapin-ratiopharm® 50 mg (Ratiopharm GmbH, Ulm, Germany) formulated as hydrophilic matrix tablets (35) were investigated. As a matrix forming agent, hypromellose (HPMC) - Methocel® K100M, K15M and K4M (Colorcon, Dartford Kent, UK), was applied in the composition of all investigated products.

### Determination of HPMC Content and Type

The HPMC content was quantified by phenol-sulphuric acid assay (20). The kinematic viscosity of dissolved tablet in 50 ml water was determined by using capillary viscometer (Ubbelohde viscometer types 50,113/Ic and 50,110/I) at 25°C. The type of HPMC was estimated using the linear regression of relationship between 8th root of kinematic viscosity and reference products (Methocel® K100 M, K15M and K4M) based on Philipoff equation η^1/8^ = (KC +1), where η is viscosity in centistokes (cSt), C is concentration of HPMC in mg/ml and K is constant specific for each molecular weight of HPMC. Due to the assignment of HPMC to only 3 types of HPMC, the effect of other excipients in the tablet was neglected.

### Dissolution Study

Since investigated extended-release hydrophilic matrix tablets are intended to release most of the active substance in the intestinal environment, USP phosphate buffer solution pH of 6.8 was used as the main dissolution medium. Dissolution testing was conducted using an USP Apparatus II with a paddle agitation speed of 50, 100 or 150 rpm (VK 7000, VanKel Industries, NJ, USA) in 900 mL dissolution medium under sink condition (26). Dissolution studies were performed in triplicate.

### Hydration and Erosion

Tablets were weighed (initial weight) and placed in 900 ml phosphate buffer pH 6.8 and agitated during the dissolution test (paddle agitation speed 50, 100 or 150 rpm), and were withdrawn from dissolution vessels at 1, 2, 4 and 6 h, weighted (wet weight), dried at 105°C overnight and reweighed (dry weight). The experiments were performed in triplicate.

$$ \boldsymbol{hydration}\ \left(\%\right)=\frac{\boldsymbol{wet}\ \boldsymbol{weight}-\boldsymbol{dry}\ \boldsymbol{weight}}{\boldsymbol{dry}\ \boldsymbol{weight}}\cdotp \mathbf{100} $$

$$ \boldsymbol{weight}\ \boldsymbol{loss}\ \left(\%\right)=\frac{\boldsymbol{initial}\ \boldsymbol{weight}-\boldsymbol{dry}\ \boldsymbol{weight}}{\boldsymbol{initial}\ \boldsymbol{weight}}\cdotp \mathbf{100} $$

### Determination of Gel Strength upon Hydration

**Fig. 1:**
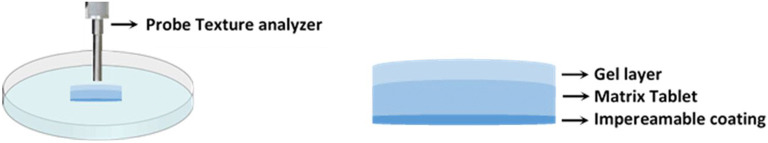
Schematic representation of gel strength determination upon hydration.

One planar base of the tablet was deep-coated with impermeable Eudragit® RS dissolved in isopropanol and subsequently glued with the covered side of the tablet to the bottom of a small petri-dish (diameter 30 mm). These samples were placed into dissolution paddle apparatus filled with 900 ml phosphate buffer pH 6.8 and stirred with paddle at 50 or 150 rpm. The gel strength of swollen tablets at predetermined time intervals was measured using a texture analyzer (TA.XTplus, Stable Micro Systems Ltd., UK) equipped with a 2 mm in diameter flat-tipped, round steel probe (Fig. 1). The test conditions where pre-test speed was 0.2 mm/s, trigger force – 0.1 g, and test speed – 0.1 mm/s. Gel strength was calculated as the ratio between the penetrating force and the displacement of the probe inside the gel according to the following equation:

$$ \boldsymbol{G}=\frac{\boldsymbol{F}}{\boldsymbol{x}}\cdotp \frac{\mathbf{1}}{{\boldsymbol{r}}_{\boldsymbol{P}}}\cdotp \mathbf{0.0098} $$

where, *G* – gel strength (MPa), *F* – force (g) registered at probe penetration, *x* – penetration depth (mm), *r*_*p*_ – radius of the probe (1 mm). The gel strength at the gel-solution interface was considered as the first point after the probe was in full contact with the gel (trigger force reached) and the initial noise disappeared. The averaged gel strength was calculated as mean of 5 consequent points (35).

## Results

All five investigated commercial products contained hypromellose (HPMC) as a matrix forming agent. However, the behavior of the tablet upon contact with dissolution medium *in vitro* or gastrointestinal fluid *in vivo* strongly depends on type and amount of HPMC. Therefore, to understand differences in release behavior, tablets were characterized in their dimension and analyzed in term of type/amount of HPMC (Table I).

**Table I:** Characteristics of Commercial HPMC Based Matrix Tablets

Product	API (dose)	HPMC			Other relevant excipinets	Tablet weight (mg)/Hardness, N	Diameter^2^ or Length^3^/Width^3^, mm	Drug loading, %
		%w/w	Type	η^1^, dL/g				
Glucophage® XR	Metformin (500 mg)	40	K100M	11.01	MCC, NaCMC	1050/177	19/9	61.1
Alfuzosin-ratiopharm® uno	Alfuzosin (10 mg)	> 70	K15M	8.98	Lactose, Povidone	308/125	9	3.63
Tromphyllin® retard	Theophylline (300 mg)	20			–	365/94	14/6	82.2
Preductal® MR	Trimetazidine (35 mg)	37	K4M	7.37	CaHPO4. 2H_2_O, Povidone	205/129	8	21.8
Quetiapin-ratiopharm®	Quetiapine (50 mg)	25			MCC, Sodium citrate	519/390	17/7	11.1

1 – intrinsic viscosity, 2 – for round tablets, 3 – for oval tablets

**Fig. 2:**
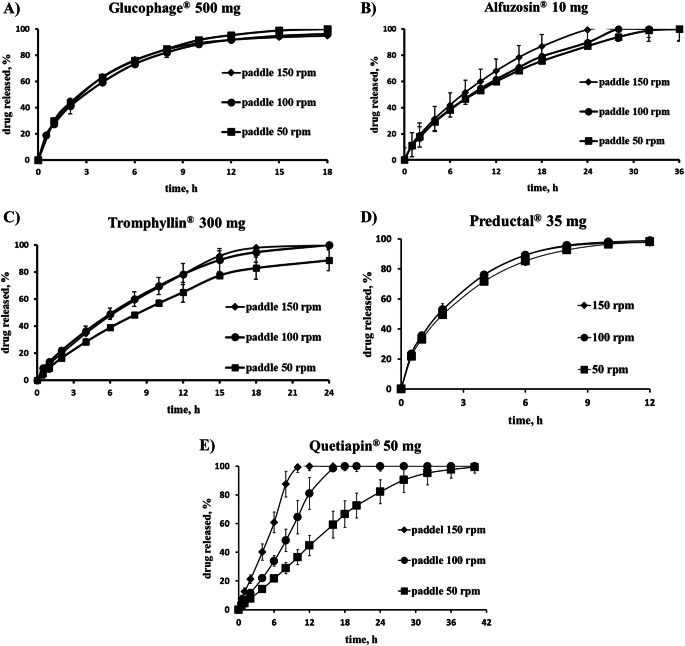
Dissolution profiles for different products at different agitation speed: (**A**) Glucophage®, (**B**) Alfuzosin®, (**C**) Tromphyllin®, (**D**) Preductal®, (**E**) Quetiapin®.

Release profiles were not affected by agitation speed in range 50–150 rpm in case of Glucophage®, Preductal® and Alfuzosin® (up to 8 h), but increased for Tromphyllin® (increasing stirring speed from 50 to 100 rpm) and markedly for Quetiapin® (Fig. 2). Visual observation of Quetiapin® tablets after release at paddle speed of 50 rpm showed that the residue of the HPMC matrix tablet was a small soft gel mass which fell apart when touched.

**Fig. 3:**
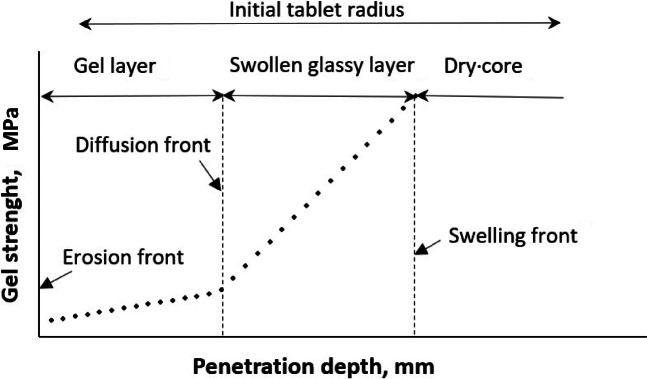
Schematic representation of gel strength profile within swellable matrix tablet.

Hydrated matrix tablets were characterized using a texture analyzer at different times of dissolution. Three regions in a swollen matrix tablet according to the degree of hydration and therefore the mechanical properties, can be identified using the texture analyzer: gel layer (highest hydration), swollen glassy layer (low hydration) and dry core (no hydration) as schematically shown in Fig. 3. The gel strength was the lowest at the gel-solution boundary, but gradually increased towards the center of the tablet. Followed partially by the hydrated region (swollen glassy layer) which is characterized by continuous subsided increase of the gel strength until the dry core is reached (Fig. 3).

**Fig. 4:**
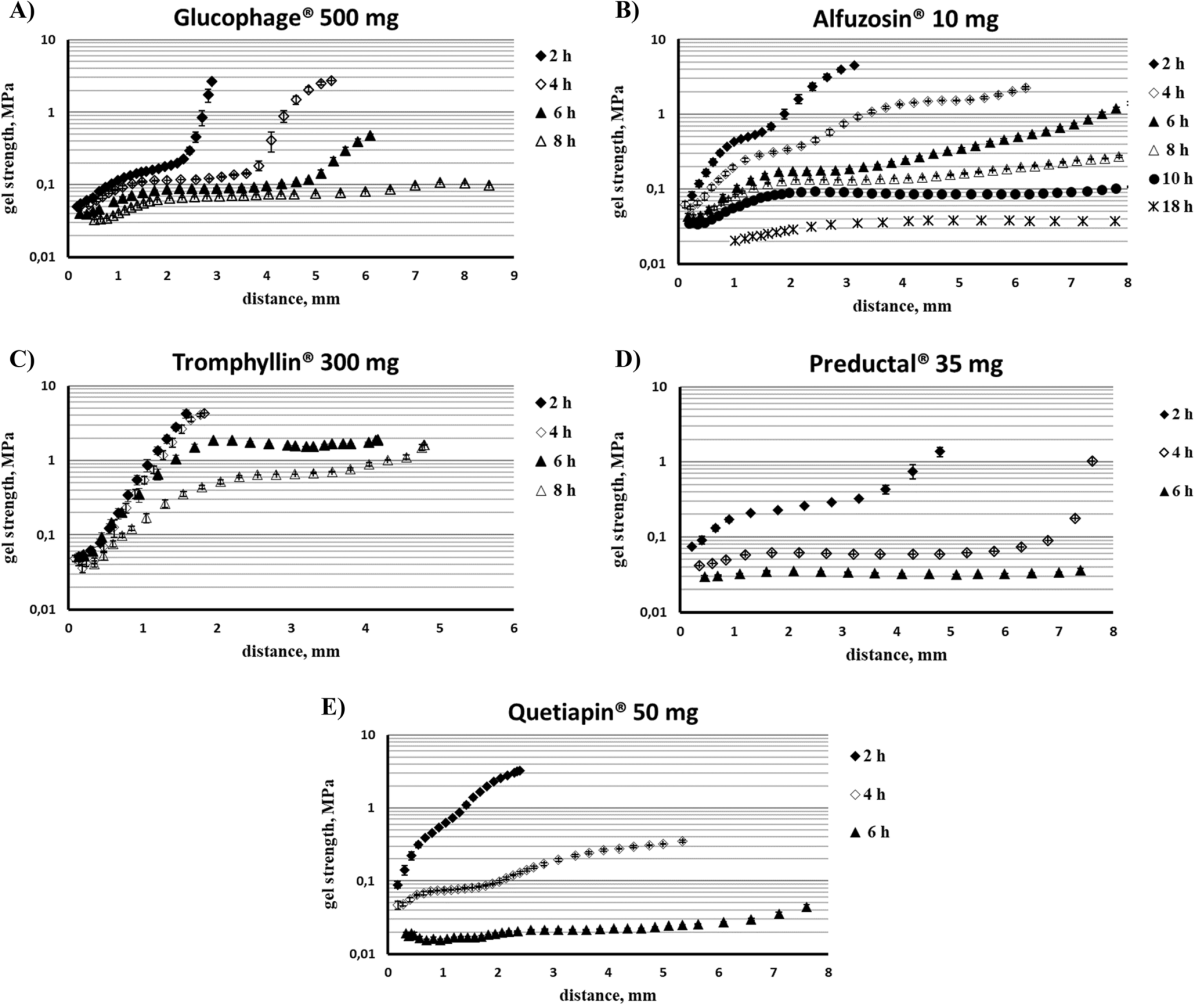
Gel strength profiles of investigated products by texture analyzer in different swelling time (in pH 6.8 with paddle apparatus at 50 rpm: (**A**) Glucophage®, (**B**) Alfuzosin®, (**C**) Tromphyllin®, (**D**) Preductal®, (**E**) Quetiapin®.

Generally, all investigated HPMC-based matrix tablets behaved similarly upon hydration and all three regions (gel layer, swollen glassy layer and dry core) could be clearly detected. However, all regions were more pronounced for Glucophage® and Preductal® tablets (Fig. 4A and D). The gel strength *vs*. penetration distance profiles of Tromphyllin® begins with a short plateau before linear increase in gel strength (Fig. 4C). In this case, a thin but detectable, uniformly hydrated layer formed due to the low amount (20%) of HPMC in the formulation. The transition from the gel layer to the swollen glassy layer became less pronounced with the progressed dissolution time, especially for Alfuzosin® and Quetiapin® (Fig. 4B and E).

**Fig. 5:**
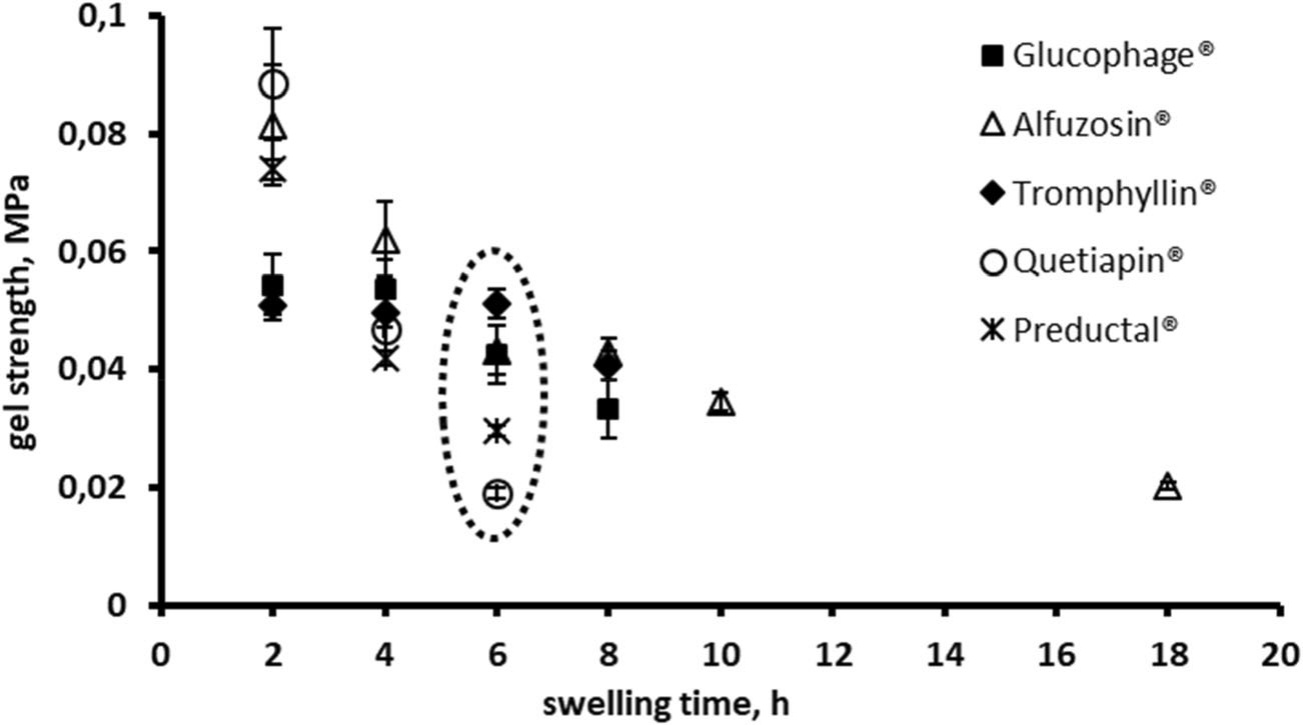
Gel strength (MPa) at gel-solution interface tested in phosphate buffer solution pH 6.8 at paddle speed of 50 rpm.

Thus, the gel strength at the erosion front was 50–90 KPa and decreased continuously over the dissolution time for all investigated tablets (Fig. 5). Interestingly, the gel strength for Quetiapin® matrix tablet was higher at early phases, e.g. 2 h, but towards 6 h dropped to the lowest values of approx. 20 KPa (Fig. 5).

**Fig. 6:**
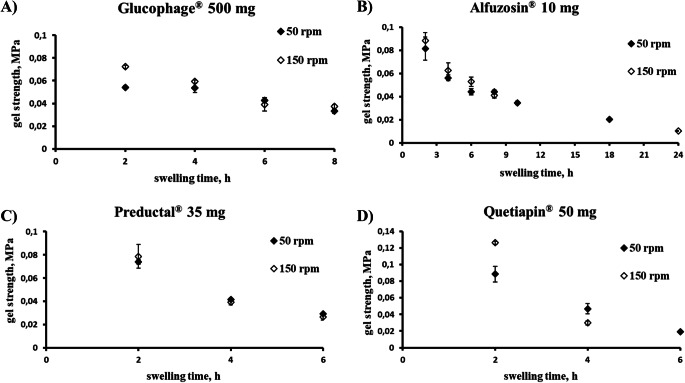
Effect of stirring speed at 50 rpm vs. 150 rpm on gel strength at gel-solution interface (MPa) in different swelling times: (**A**) Glucophage®, (**B**) Alfuzosin®, (**C**) Preductal®, (**D**) Quetiapin®. Commercial product Tromphyllin® was not available for this experiment.

For all investigated products, tendentially, the higher gel strength was registered at higher stirring speeds (Fig. 6), probably, due to a thinner gel layer remaining on the surface of tablets at higher stirring speed. In this case, the mechanical properties of the swollen glassy layer have a stronger impact on the determined gel strength. Nevertheless, this difference was negligible for Glucophage®, Alfuzosin® and Preductal®, but not for Quetiapin® (Fig. 6 A-C *vs*. D). In the case of Quetiapin®, a stronger drop of gel strength occurred upon stirring at 150 *vs*. 50 rpm (Fig. 6D).

**Fig. 7:**
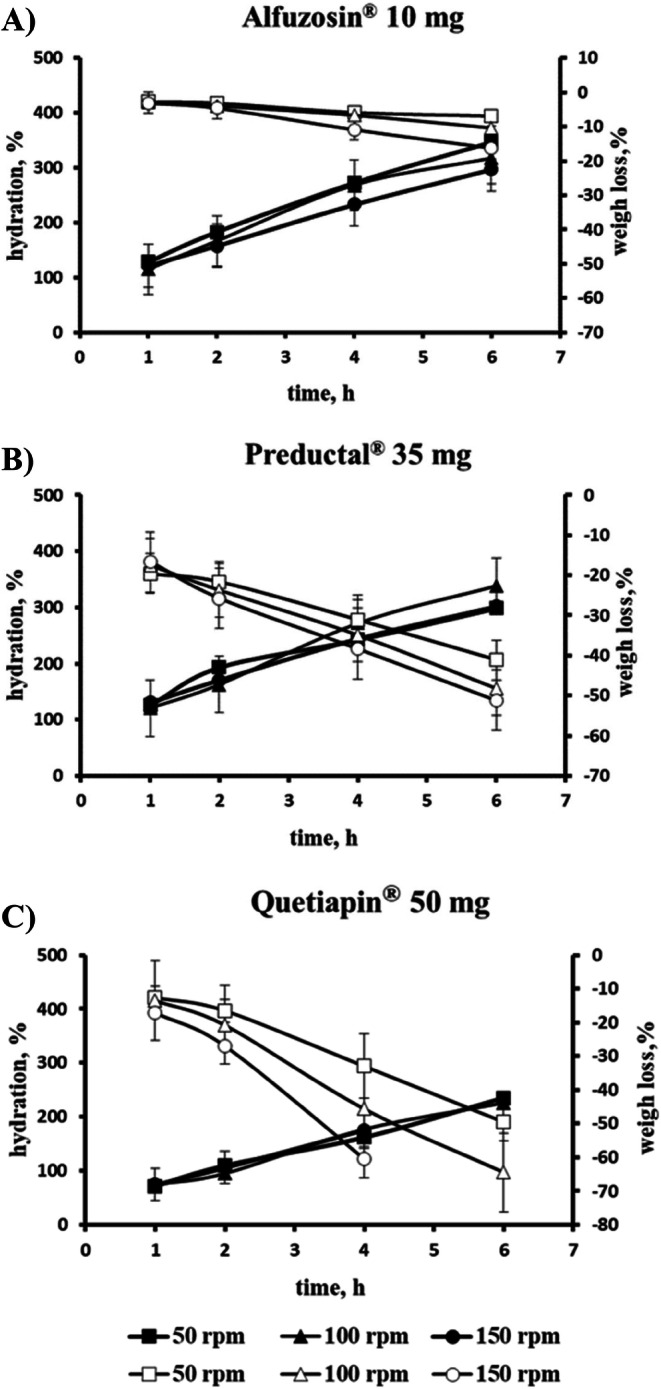
Hydration (closed symbols) and weight loss (opened symbols) of matrix tablets during dissolution pH 6.8 at different agitation speed: (**A**) Alfuzosin®, (**B**) Preductal®, (**C**) Quetiapin®. Commercial product Tromphyllin® and Glucophage® were not available for this experiment.

The hydration is mainly determined by the properties of the matrix (amount and property of matrix forming polymer) and release medium. Therefore, the hydration of investigated tablets increased gradually with the dissolution time, while it was almost independent of stirring speed (Fig. 7).

The erosion, characterized by weight loss of solids during dissolution, is mainly determined by gel strength and mechanical stress. It increased over the dissolution time and differed depending on the stirring speed. Thus, the slope of declining curves “weight loss – time” was barely affected by stirring speed for Alfuzosin® tablets (Fig. 7A). For Preductal® tablets, the mentioned slope, and its susceptibility to stirring speed was higher (Fig. 7B). The highest slope was for Quetiapin® tablets which also strongly increased with increased stirring speed (Fig. 7C).

## Discussion

The difference in susceptibility to mechanical stress of investigated commercial products based on hydrophilic matrix tablets (26) can be well explained by the physical properties of tablets in the media during *in vitro* drug release. The drug release rate is dependent on the movement of different fronts and consequently strongly dependent on the interlay of the erosion and diffusion front (1,11,15).

Therefore, drug release from such tablets is strongly associated with the gel strength and, thus, with intensity of erosion and susceptibility to mechanical stress. While the gel strength upon hydration depends on the type, molecular weight, amount of matrix forming polymer in the tablet, drug solubility and presence of other excipients.

Robust products demonstrated a relatively high gel strength (Fig. 5). E.g. freely soluble metformin hydrochloride (Table II) was formulated in Glucophage® with approx. 40% of high viscosity HPMC of type K100M, sodium carboxymethyl cellulose (NaCMC) and microcrystalline cellulose (MCC) (Table I). It is worth to mention that besides the matrix former, other formulation ingredients (e.g. drug and filler) are counted for in the formation of a stronger gel layer in these formulations. For example, it was shown that a combination of HPMC with NaCMC, e.g. in Glucophage®, has two effects. Firstly, rheological synergism of HPMC and NaCMC results in prolongation of the release profile (42), and secondly, the complex formation between anionic polymer NaCMC and cationic drug metformin mitigates rapid initial release of the highly soluble drug and retardation of drug release (43). Regarding the effect of the filler, domains of microcrystalline cellulose remain within the hydrating matrix, which results in weak physical cross-linking between MCC and HPMC, and thus increased gel strength (43).

**Table II:** Solubility of APIs

API	pH of medium	Solubility mg/ml	Descriptive Term	Ref.
Metformin hydrochloride	6.7	307.9	freely	(36,37)
Alfuzosin hydrochloride	6.6	235	freely	(38)
Theophylline	6.8	11.6	slightly	(39)
Trimetazidine hydrochloride	6.7	340	freely	(40)
Quetiapine fumarate	6.8	1.0	slightly	(41)

Other robust formulation of freely soluble - alfuzosin hydrochloride (Table I) was formulated with >70% K15M (Alfuzosin®) and demonstrated relatively high gel strength (Fig. 5) with an erosion not strongly depended on the agitation speed (Fig. 7A). This agrees with previous studies that the erosion rate *in vitro* and *in vivo* decrease with increasing fraction of a high molecular weight HPMC in tablets above the reported values for polymer percolation threshold of 30–35% *w*/w (21,44). Also Glucophage® and Alfuzosin® were relatively robust against biorelevant mechanical stress when a loading force 2 N was applied after 1, 2 or 4 h of dissolution (26).

For Preductal® tablets, the gel strength and erosion were almost independent on agitation speed (Fig. 6C and 7B, respectively) and varying the stirring speed in the investigated range did not affect the drug release (Fig. 2D). However, the effect of biorelevant mechanical stress was significant (26).

In contrast, slightly soluble quetiapine (Table II) formulated with only approx. 25% HPMC K 4 M in the Quetiapin® (Table I) demonstrated non-robust drug release (Fig. 2E). Undissolved quetiapine particles in the gel layer could be a reason for reduced entanglement of polymer chains and low robustness of the tablets (16). Moreover, quetiapine is a weakly basic drug and was formulated with sodium citrate to maintain sufficient solubility in the higher pH of the intestine. Based on a Hofmeister effect, sodium citrate can reduce swelling of HPMC particles and formation of continuous gel, and thus, accelerate erosion (45). In fact, the erosion was very pronounced (Fig. 7C) being the predominant release mechanism for Quetiapin® matrix tablets. The gel layer of Quetiapin® tablets demonstrated rapid decrease of gel strength upon hydration (Fig. 5) which was strongly dependent on agitation speed (Fig. 6D). Therefore, quetiapine matrix tablets were most affected by biorelevant mechanical stress resulted in high variations of plasma concentrations profiles (26).

## Conclusion

Our findings clearly demonstrated that drug release from tablets formulated with higher molecular weight HPMC and with content ≥20% *w*/w was robust against a wide range of agitation speeds (50–150 rpm) and applied biorelevant mechanical stress during *in vitro* release. This could be attributed to sufficient gel strength ≥20 KPa up to 6 h of hydration. These findings may provide deeper insight for formulation scientists to select formulation components more properly to achieve more robust dosage forms.

## ABBREVIATIONS

GI: Gastrointestinal
HPMC: Hydroxypropyl methylcellulose (hypromellose)
NaCMC: Sodium carboxymethyl cellulose
MCC: Microcrystalline cellulose

## References

[1] Colombo P, Bettini R, Peppas NA (1999). Observation of swelling process and diffusion front position during swelling in hydroxypropyl methyl cellulose (HPMC) matrices containing a soluble drug. J Control Release.

[2] Kavanagh N, Corrigan OI (2004). Swelling and erosion properties of hydroxypropylmethylcellulose (Hypromellose) matrices--influence of agitation rate and dissolution medium composition. Int J Pharm.

[3] Rajabi-Siahboomi AR, Bowtell RW, Mansfield P, Henderson A, Davies MC, Melia CD (1994). Structure and behaviour in hydrophilic matrix sustained release dosage forms: 2. NMR-imaging studies of dimensional changes in the gel layer and core of HPMC tablets undergoing hydration. J Control Release.

[4] Colombo P, Bettini R, Santi P, de Ascentiis A, Peppas NA (1996). Analysis of the swelling and release mechanisms from drug delivery systems with emphasis on drug solubility and water transport. J Control Release.

[5] Colombo P, Bettini R, Catellani PL, Santi P, Peppas NA (1999). Drug volume fraction profile in the gel phase and drug release kinetics in hydroxypropylmethyl cellulose matrices containing a soluble drug. Eur J Pharm Sci.

[6] Timmins P, Pygall SR, Melia CD. Hydrophilic matrix tablets for oral controlled release. 2014. USA: Springer.

[7] Ju RT, Nixon PR, Patel MV (1995). Drug release from hydrophilic matrices. 1. New scaling laws for predicting polymer and drug release based on the polymer disentanglement concentration and the diffusion layer. J Pharm Sci.

[8] Korner A (2005). Molecular information on the dissolution of polydisperse polymers: mixtures of long and short poly(ethylene oxide). J Phys Chem B.

[9] Korner A (2009). Influence of different polymer types on the overall release mechanism in hydrophilic matrix tablets. Molecules.

[10] Turner S, Federici C, Hite M, Fassihi R (2004). Formulation development and human in vitro-in vivo correlation for a novel, monolithic controlled-release matrix system of high load and highly water-soluble drug niacin. Drug Dev Ind Pharm.

[11] Peppas NA, Narasimhan B (2014). Mathematical models in drug delivery: how modeling has shaped the way we design new drug delivery systems. J Control Release.

[12] Vrentas JS, Jarzebski CM, Duda JL (1975). A Deborah number for diffusion in polymer-solvent systems. AICHE J.

[13] Ritger PL, Peppas NA (1987). A simple equation for description of solute release I. Fickian and non-fickian release from non-swellable devices in the form of slabs, spheres, cylinders or discs. J Control Release.

[14] Ritger PL, Peppas NA (1987). A simple equation for description of solute release II. Fickian and anomalous release from swellable devices. J Control Release.

[15] Yin X, Li H, Guo Z, Wu L, Chen F, de Matas M, Shao Q, Xiao T, York P, He Y, Zhang J (2013). Quantification of swelling and erosion in the controlled release of a poorly water-soluble drug using synchrotron X-ray computed microtomography. AAPS J.

[16] Bettini R, Catellani PL, Santi P, Massimo G, Peppas NA, Colombo P (2001). Translocation of drug particles in HPMC matrix gel layer: effect of drug solubility and influence on release rate. J Control Release.

[17] Siepmann F, Karrout Y, Gehrke M, Penz FK, Siepmann J (2017). Limited drug solubility can be decisive even for freely soluble drugs in highly swollen matrix tablets. Int J Pharm.

[18] Maderuelo C, Zarzuelo A, Lanao JM (2011). Critical factors in the release of drugs from sustained release hydrophilic matrices. J Control Release.

[19] Sako K, Sawada T, Nakashima H, Yokohama S, Sonobe T (2002). Influence of water soluble fillers in hydroxypropylmethylcellulose matrices on in vitro and in vivo drug release. J Control Release.

[20] Ghori MU, Ginting G, Smith AM, Conway BR (2014). Simultaneous quantification of drug release and erosion from hypromellose hydrophilic matrices. Int J Pharm.

[21] Jain AK, Söderlind E, Viridén A, Schug B, Abrahamsson B, Knopke C, Tajarobi F, Blume H, Anschütz M, Welinder A, Richardson S, Nagel S, Abrahmsén-Alami S, Weitschies W (2014). The influence of hydroxypropyl methylcellulose (HPMC) molecular weight, concentration and effect of food on in vivo erosion behavior of HPMC matrix tablets. J Control Release.

[22] Feely LC, Davis SS (1988). The influence of polymeric excipients on drug release from hydroxypropylmethylcellulose matrices. Int J Pharm.

[23] Narasimhan B, Peppas NA (1997). Molecular analysis of drug delivery systems controlled by dissolution of the polymer carrier. J Pharm Sci.

[24] Jamzad S, Tutunji L, Fassihi R (2005). Analysis of macromolecular changes and drug release from hydrophilic matrix systems. Int J Pharm.

[25] Kamba M, Seta Y, Kusai A, Nishimura K (2002). Comparison of the mechanical destructive force in the small intestine of dog and human. Int J Pharm.

[26] Mohylyuk V, Goldoozian S, Andrews GP, Dashevskiy A (2020). IVIVC for extended release hydrophilic matrix tablets in consideration of biorelevant mechanical stress. Pharm Res.

[27] Klancar U (2013). A novel beads-based dissolution method for the in vitro evaluation of extended release HPMC matrix tablets and the correlation with the in vivo data. AAPS J.

[28] Abrahamsson B, Roos K, Sjogren J (1999). Investigation of prandial effects on hydrophilic matrix tablets. Drug Dev Ind Pharm.

[29] Ghimire M, Hodges LA, Band J, O'Mahony B, McInnes FJ, Mullen AB, Stevens HNE (2010). In-vitro and in-vivo erosion profiles of hydroxypropylmethylcellulose (HPMC) matrix tablets. J Control Release.

[30] Klancar U, Horvat M, Baumgartner S (2012). Correlating cellulose derivative intrinsic viscosity with mechanical susceptibility of swollen hydrophilic matrix tablets. AAPS PharmSciTech.

[31] Kamba M, Seta Y, Kusai A, Ikeda M, Nishimura K (2000). A unique dosage form to evaluate the mechanical destructive force in the gastrointestinal tract. Int J Pharm.

[32] Garbacz G, Klein S (2012). Dissolution testing of oral modified-release dosage forms. J Pharm Pharmacol.

[33] Bellmann S, Lelieveld J, Gorissen T, Minekus M, Havenaar R (2016). Development of an advanced in vitro model of the stomach and its evaluation versus human gastric physiology. Food Res Int.

[34] Garbacz G, Wedemeyer RS, Nagel S, Giessmann T, Mönnikes H, Wilson CG, Siegmund W, Weitschies W (2008). Irregular absorption profiles observed from diclofenac extended release tablets can be predicted using a dissolution test apparatus that mimics in vivo physical stresses. Eur J Pharm Biopharm.

[35] Xiao L (2007). Effect of gel strength on drug release from Swellable matrices through polymer Erosion.

[36] Desai D, Wong B, Huang Y, Ye Q, Tang D, Guo H, Huang M, Timmins P (2014). Surfactant-mediated dissolution of metformin hydrochloride tablets: wetting effects versus ion pairs diffusivity. J Pharm Sci.

[37] Desai D, Wong B, Huang Y, Ye Q, Guo H, Huang M, Timmins P (2015). Wetting effects versus ion pairs diffusivity: interactions of anionic surfactants with highly soluble cationic drugs and its impact on tablet dissolution. J Pharm Sci.

[38] Alfuzosin HCl ER. 10mg tablets (Sanofi Synthelabo; Appl. #21-287) Clinical Pharmacology and Biopharmaceutics Review. 2002, FDA. CDER USA.

[39] Serajuddin AT, Jarowski CI (1985). Effect of diffusion layer pH and solubility on the dissolution rate of pharmaceutical acids and their sodium salts. II: salicylic acid, theophylline, and benzoic acid. J Pharm Sci.

[40] Mohylyuk V, Davtian L (2015). Effect of diluent types and soluble diluents particle size on the dissolution profile of trimetazidine dihydrochloride and caffeine from Kollidon SR matrix tablets. Int J Pharmtech Res.

[41] Volgyi G (2010). Study of pH-dependent solubility of organic bases. Revisit of Henderson-Hasselbalch relationship. Anal Chim Acta.

[42] Palmer D, Levina M, Nokhodchi A, Douroumis D, Farrell T, Rajabi-Siahboomi A (2011). The influence of sodium carboxymethylcellulose on drug release from polyethylene oxide extended release matrices. AAPS PharmSciTech.

[43] Dürig T, Fassihi R (2002). Guar-based monolithic matrix systems: effect of ionizable and non-ionizable substances and excipients on gel dynamics and release kinetics. J Control Release.

[44] Using Dow excipients for controlled release of drugs in hydrophilic matrix systems 2006, Dow Chemicals: USA.

[45] Pygall SR, Kujawinski S, Timmins P, Melia CD (2009). Mechanisms of drug release in citrate buffered HPMC matrices. Int J Pharm.

